# To Dr. Batty

**Published:** 1807-01

**Authors:** J. Rogers

**Affiliations:** St. Petersburgh


### To Dr. BATTY.

Dear Sir,

INCLOSED I have sent you the copy of a Report made to me by a young Surgeon in my department, of the effect of lightning on some workmen employed at Cronstadt on the 17th of last month; and request that you will cause it to be inserted in the Medical Journal.

I am, &c.

*St. Petersburgh, Sept.* 9, 1806. J. ROGERS.

---

Die vjto. mensis Augusti, 1806to anno, horâ decimâ vespertinâ, quo die officio diarii fungebar, jussu Domini Doctoris Lackmann, missus eram ad saxarios fulgore tactos, ut illis auxilium praeberem, et rem strenue perquirerem. Cameram ingressus, qua laborantes habitarunt, sequentia inveni: duo ex attactis morte momentaneâ correpti pavimento prostrati jacebant, reliqui superstites graviter cruciebantur, eorumque unus, ictu electrico validâ commotione cerebri percussus, motus involuntarios producebat ac delirabat, alter de conquassatione pectoris querebatur, tertius utramque manum paralyticam totumque corpus sat insigniter affectum praebebat. Duo adhuc praesenter levem solummodo commotionem perpessi magis terrore quam fulmine concussi inveniebantur.

Quod ad habitum externum mortuorum, nulla signa eximia aparuerunt, nisi ut capilli in occipite fulmine consumpti fuerant. Altero vero die totum corpus serpentino modo sugillationibus sanguinis coloratum, venae jugulares turgidae

---

turgidae, collum lividum, abdomen tumidum et durum, unde apoplexiâ mortem conciliatam fuisse. Intumescentia autem abdominis extravasatione sanguinis rupturâ vasorum orta mihi videtur.

Investigando alternatim alios, imprimis eum qui commotionem cerebri passus erat et vulnus palpebrae superioris oculi dextri contusione acceperat, statim exui et omnia vestamenta detrahi jussi, et embrocationes capitis ex aqua glaciali cum anaticâ parte aceti institui; intus nihil porrexi, cum nulla ad manus medicamenta habui. Ceteris laborantibus frictiones e cremato simplici ad partes adfectas feci.

Praeterlapso unius horae spatio laborantes in nosocomium deduxi, iisque palatiis posui, quibus ipsemet curae aegrorum prospicio. Ibi sequentia ordinavi; illi qui commotionem pectoris passus erat, venam secavi et sanguinis ℔j. detraxi, quum excretione sputorum sanguine mixtorum plethoram adesse censui; illi qui commotione cerebri laboravit, abscissis capillis capitis, fomentationes ex aceto camphorato per totam noctem institui, et ad nucham surasque sinapismata fortia apposui; illi autem cuius manus paralyticos inveneram, frictiones e balsamo saponato adhibui. Interne ordinavi:

℞. Infusi herbae arnicae ℥viij.

Spiritus Mindereri ℥iij.

Mellis despumati ℥ij.

M. capiat aeger omni horâ cochlear mensuale unum.

Vulnus palpebrae plumaceolâ aquâ vegeto-minerali imbutâ deligavi.

Altero die, matutino tempore, duo, qui levem concussione corporis perpessi erant, sani ad obeundos labores pristinos nosocomio egrediebantur; reliqui debilitate laborantes adhuc nosocomio retinentur.

CANDIDATUS CHIRURGIAE PETROFF.

